# Protein kinase C activation disrupts epithelial apical junctions via ROCK-II dependent stimulation of actomyosin contractility

**DOI:** 10.1186/1471-2121-10-36

**Published:** 2009-05-07

**Authors:** Andrei I Ivanov, Stanislav N Samarin, Moshe Bachar, Charles A Parkos, Asma Nusrat

**Affiliations:** 1 Department of Medicine, University of Rochester, Rochester, NY 14642, USA; 2 Department of Pathology and Laboratory Medicine, Emory University, Atlanta, GA, 30322, USA

## Abstract

**Background:**

Disruption of epithelial cell-cell adhesions represents an early and important stage in tumor metastasis. This process can be modeled *in vitro *by exposing cells to chemical tumor promoters, phorbol esters and octylindolactam-V (OI-V), known to activate protein kinase C (PKC). However, molecular events mediating PKC-dependent disruption of epithelial cell-cell contact remain poorly understood. In the present study we investigate mechanisms by which PKC activation induces disassembly of tight junctions (TJs) and adherens junctions (AJs) in a model pancreatic epithelium.

**Results:**

Exposure of HPAF-II human pancreatic adenocarcinoma cell monolayers to either OI-V or 12-O-tetradecanoylphorbol-13-acetate caused rapid disruption and internalization of AJs and TJs. Activity of classical PKC isoenzymes was responsible for the loss of cell-cell contacts which was accompanied by cell rounding, phosphorylation and relocalization of the F-actin motor nonmuscle myosin (NM) II. The OI-V-induced disruption of AJs and TJs was prevented by either pharmacological inhibition of NM II with blebbistatin or by siRNA-mediated downregulation of NM IIA. Furthermore, AJ/TJ disassembly was attenuated by inhibition of Rho-associated kinase (ROCK) II, but was insensitive to blockage of MLCK, calmodulin, ERK1/2, caspases and RhoA GTPase.

**Conclusion:**

Our data suggest that stimulation of PKC disrupts epithelial apical junctions via ROCK-II dependent activation of NM II, which increases contractility of perijunctional actin filaments. This mechanism is likely to be important for cancer cell dissociation and tumor metastasis.

## Background

Progression and dissemination of epithelial tumors is accompanied by a loss of morphological features of epithelial cells and acquisition of mesenchymal cell phenotype known as epithelial to mesenchymal transition (EMT) [1,2]. Weakening and disruption of intercellular adhesions represents one of the most characteristic features of EMT [3,4]. Differentiated epithelial cells strongly adhere to each other via specialized junctional complexes assembled at the lateral plasma membrane [5-7]. Among them, the most apically-located tight junctions (TJs) and adherens junctions (AJs) are critical for epithelial cell differentiation and maintenance of the integrity of epithelial layers [5-7]. TJs and AJs mediate cell-cell adhesions through homotypical interactions of their transmembrane proteins such as occludin, claudins and E-cadherin [5-7]. Furthermore, these junctional complexes are affiliated with the apical actin cytoskeleton, and participate in outside in transduction of signals and forces [5,8].

Disruption of TJs and AJs occurs at the early stage of EMT and has two major functional consequences in tumor cells. One is the increase in cell proliferation, and another is enhanced cell motility [3,4]. The former reflects the fact that TJs and AJs sequester many transcriptional regulators such as β-catenin, ZONAB, and symplekin, which upon junctional disassembly translocate into the nucleus to stimulate expression of genes controlling cell division [9,10]. The later effect is due to dramatic cytoskeletal reorganizations induced by the loss of intercellular contacts and resulting in altered cell-matrix adhesions and actin filament dynamics [11-13]. Although TJ/AJ disassembly plays an important role in tumor growth and metastasis, its molecular mechanisms remain poorly investigated.

Disruption of epithelial junctions during EMT is commonly modeled *in vitro *by exposing epithelial cells to growth factors or chemical tumor promoters [2,14]. Among them, carcinogens targeting protein kinase C (PKC) are the most extensively characterized. PKC, which plays a key role in cancer signaling pathways, is dramatically stimulated by two major classes of pharmacological agents: phorbol esters and indole alkaloids, teleocidins [15,16]. These PKC activators elicit a variety of responses characteristic of tumor cells, including stimulation of cell proliferation, decreased sensitivity to apoptosis, increased cell-matrix adhesion and cell migration/invasion [17,18]. Because of this, phorbol esters and teleocidins are widely used to study signaling pathways which underline tumor progression and metastasis.

A large body of evidence indicates that scattering/invasiveness of epithelial cells induced by PKC-targeting tumor promoters involves disassembly of intercellular junctions. Indeed, 12-O-tetradecanoylphorbol-13-acetate (TPA) was shown to disrupt AJs in Madin-Darby canine kidney (MDCK) cells [19-21], mouse epidermal cells [22], and rat liver epithelial cells [23]. Furthermore, TPA and teleocidin have been shown to rapidly increase paracellular permeability and disassemble TJs in confluent monolayers of MDCK cells [24,25], LLC-PK1 porcine renal epithelial cells [26-28], and human corneal epithelial cells [29]. However, molecular mechanisms underlying disassembly of epithelial junctions by PKC-targeting tumor promoters remain poorly characterized. Several studies highlighted the role of endocytosis of AJ/TJ proteins E-cadherin and occludin [19,20,22,25]. Nevertheless, endocytosis alone cannot be responsible for PKC-dependent junctional breakdown. Indeed, a continuous internalization of E-cadherin and claudins in confluent epithelial cell monolayers does not result in AJ/TJ disassembly [30-32], being antagonized by the apical actin cytoskeleton, known to associate with and stabilize AJ and TJ structure [5,8]. Reorganization/disassembly of the perijunctional actin cytoskeleton is required for the large-scale disruption and internalization of epithelial apical junctions [33-35].

PKC is a powerful regulator of the actin cytoskeleton in a variety of cells [36], and phorbol esters have been shown to induce dramatic reorganization of actin filaments in epithelial monolayers [23]. It is therefore likely that disassembly and internalization of epithelial junctions induced by PKC activators is mediated by remodeling of the perijunctional actin cytoskeleton. Reorganizations of the actin cytoskeleton are usually driven by myosin II motor, which slides actin filaments against each other, thus producing contractile forces [37,38]. Epithelial cells express nonmuscle myosin (NM) II, which is enriched in perijunctional circumferential F-actin bundles [39,40]. Furthermore, NM II activity was shown to be critical for disassembly of epithelial junctions caused by various stimuli, such as depletion of extracellular calcium and proinflammatory cytokines [35,40-43]. However, it is unknown whether NM II plays a role in the disruption of epithelial junctions during cancer cell metastasis and specifically during junctional disassembly induced by PKC-activating tumor promoters.

The aim of this study was to investigate the role of NM II in the disassembly of epithelial apical junctions caused by PKC-targeting tumor promoters, which mimic the disruption of epithelial cell-cell adhesions during EMT and tumor metastasis. We rationalized that appropriate model cell line for this study should fulfill the following criteria: 1) to be a human tumor cell line; 2) to have well-developed TJs and AJs; 3) to readily disassemble their junctions after exposure to PKC-activating tumor-promoters. However, MDCK and LLC-PK1 cell lines, which are widely used to study phorbol ester-induced junctional disruption, do not fulfill the first criteria since they are neither human, nor cancer cells. On the other hand, well characterized human colonic carcinoma cell lines, such as T84, Caco-2, and HT-29 do not respond to PKC activation by junctional disassembly [44-48]. To overcome this problem, we used HPAF-II human pancreatic adenocarcinoma cells. These cells readily differentiate to form well-defined apical junctions [49,50], which appear to be easily disruptable by tumor promoters octylindolactam (OI)-V and TPA. Using this model, we observe that OI-V-induced disassembly of epithelial AJs and TJs is mediated by activation of NM II, which is stimulated by Rho-associated kinase (ROCK) II in a RhoA-independent manner.

## Results

### OI-V and TPA rapidly disrupt paracellular barrier and induce disassembly of epithelial apical junctions

**Figure 1:**
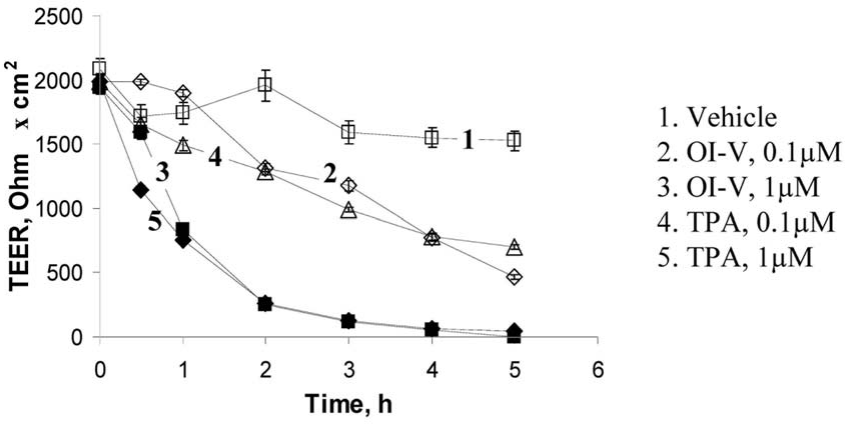
**Tumor promoters increase paracellular permeability of human pancreatic epithelial cell monolayers**. Confluent HPAF-II cell monolayers were exposed to either vehicle, or two concentrations of OI-V or TPA, and the integrity of the paracellular barrier was evaluated by transepithelial electrical resistance (TEER) measurements. Both tumor promoters induce a rapid and dose-dependent decrease in TEER, thus indicating the increase in paracellular permeability.

**Figure 2:**
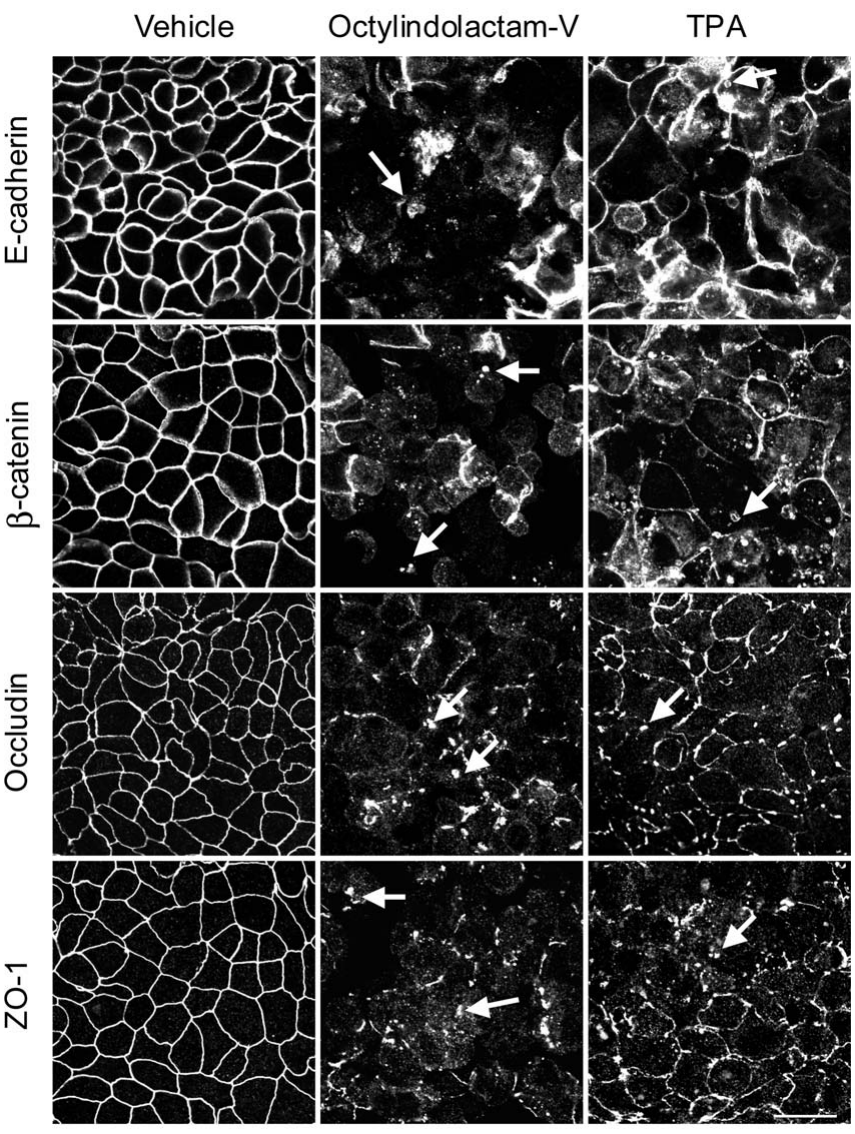
**Tumor promoters induce rapid disassembly of epithelial tight junctions and adherens junctions**. Confluent HPAF-II cell monolayers were treated for 5 h with either vehicle, OI-V, or TPA (each, 1 μM). Localization of AJ proteins (E-cadherin, β-catenin) and TJ proteins (occludin, ZO-1) was determined by fluorescence labeling and confocal microscopy. Both tumor promoters induce translocation of AJ and TJ proteins from the areas of cell-cell contact into cytosol (arrows). Bar, 20 μm.

HPAF-II cells cultured on permeable membrane support rapidly (within 5–7 days) developed confluent cell monolayers with high (1,500–2,100 Ohm × cm^2^) transepithelial electrical resistance (TEER) (Figure 1). Immunofluorescence analysis of these monolayers showed a typical 'chicken wire' labeling pattern for E-cadherin, β-catenin, occludin and ZO-1 at the cell apex (Figure 2), which is characteristic for the mature AJs and TJs. To investigate the effects of PKC activators on apical junctions, HPAF-II cells were treated with 0.1 μM and 1 μM of either OI-V or TPA. In this concentration range, both agents reportedly induce membrane translocation and activation of 50–100% of different PKC izoenzymes [51,52]. Exposure of HPAF-II cell monolayers to either OI-V or TPA resulted in a rapid and dose-dependent increase in paracellular permeability (Figure 1). Thus, 1 μM of OI-V (curve 3) or TPA (curve 5) decreased TEER from initial value of ~2000 Ohm × cm^2 ^to ~5 Ohm × cm^2 ^within 5 h. In contrast, vehicle-treated cells maintained high TEER (> 1500 Ohm × cm^2^; Figure 1, curve 1).

To analyze if the observed increase in paracellular permeability was caused by alteration in junctional structure, we investigated effects of OI-V and TPA on localization of AJ and TJ proteins in HPAF-II cells. Both agents induced dramatic reorganization of apical junctions. These reorganizations were manifested by the loss of TJ (occludin and ZO-1) and AJ (E-cadherin and β-catenin) proteins from the areas of cell-cell contacts and their accumulation in cytosolic dot-like structures (Figure 2, arrows). Interestingly, OI-V caused more profound disruption of junctional morphology than TPA (Figure 2), which is consistent with a previous study demonstrating a superior potency of teleocidin over phorbol ester in increasing permeability of LLC-PK1 cell monolayers [27]. Because of its higher efficiency, all subsequent experiments were performed using 1 μM of OI-V.

### Classical PKC isoenzymes mediate OI-V-induced junctional disassembly

Next we sought to elucidate which intracellular signal initiates OI-V-dependent junctional disassembly. While teleocidins and phorbol esters primarily signal through activation of PKC, several other target proteins, such as protein kinase D, chimaerins, and Munc 13 have been recently identified [53]. Therefore, we analyzed the role of PKC in OI-V-mediated AJ/TJ disassembly in HPAF-II cells. In addition, we sought to determine which subclass of PKC is involved. PKC isoenzymes are classified into three subclasses: "classical" (PKCα, βI, βII, and γ), "novel" (PKCδ, θ, ε and η) and "atypical" (PKCζ and ι/λ) [54,55]. Since only first two subclasses are activated by teleocidins and phorbol esters, we asked whether classical or novel PKC mediate disruption of epithelial junctions.

**Figure 3:**
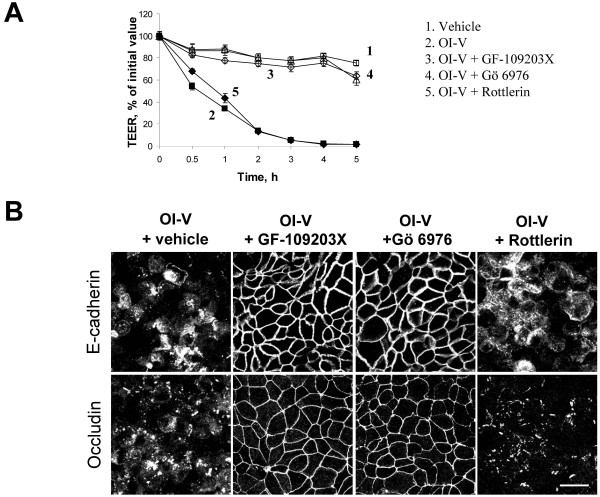
**Classical PKC isoenzymes mediate OI-V-induced disruption of epithelial apical junctions**. HPAF-II cells were treated for 5 h with either OI-V alone (1 μM) or in a combination with pharmacological inhibitors of different subfamilies of PKC isoenzymes. The barrier properties of cell monolayers were determined by TEER measurement (**A**), and the integrity of AJ and TJ was determined by immunofluorescence labeling for E-cadherin and occludin respectively (**B**). A dual inhibitor of classical and novel PKC, GF-109203X (10 μM), as well as a selective inhibitor of classical PKC, Gö 6976 (5 μM) significantly attenuate octylindiolactam-induced disruption of paracellular barrier and TJ/AJ disassembly, whereas inhibitor of a novel PKC, rottlerin (10 μM), has no effects. Bar, 20 μm.

To address these questions, we first investigated which PKC isoforms are expressed in HPAF-II cells. RT-PCR and immunoblotting analyses revealed the presence of multiple isoforms including three classical (PKCα, β I, and γ) and four novel (PKCδ, θ, ε and η) isoenzymes (Additional Files 1 &2). In OI-V treated cells, members of both classical (PKCα and βI) and novel (PKCδ) subclasses rapidly (within 1 h) accumulated at cell membranes (Additional File 2), which is indicative of PKC activation [51,52]. To gain insight into the functional roles of different PKC subclasses in OI-V-mediated junctional disassembly, pharmacological PKC inhibitors with different subclass specificity were used. They include GF-109203X, which inhibits both classical and novel PKC [56,57], a selective inhibitor of classical PKC, Gö 6976 [58], and an inhibitor of novel PKC δ and θ, rottlerin [59,60]. Incubation of HPAF-II cells with either GF-109203X (Figure 3A, curve 3) or Gö 6976 (curve 4) prevented OI-V-induced decrease in TEER, whereas rottlerin (curve 5) had no effect. Furthermore, GF-109203X and Gö 6976, but not rottlerin blocked OI-V-induced AJ/TJ disassembly (Figure 3B). These data suggest that activation of classical PKC triggers disruption of the paracellular barrier and breakdown of apical junctions in OI-V-treated pancreatic epithelial cells.

### OI-V-induced disassembly of epithelial junctions is mediated by NM II

**Figure 4:**
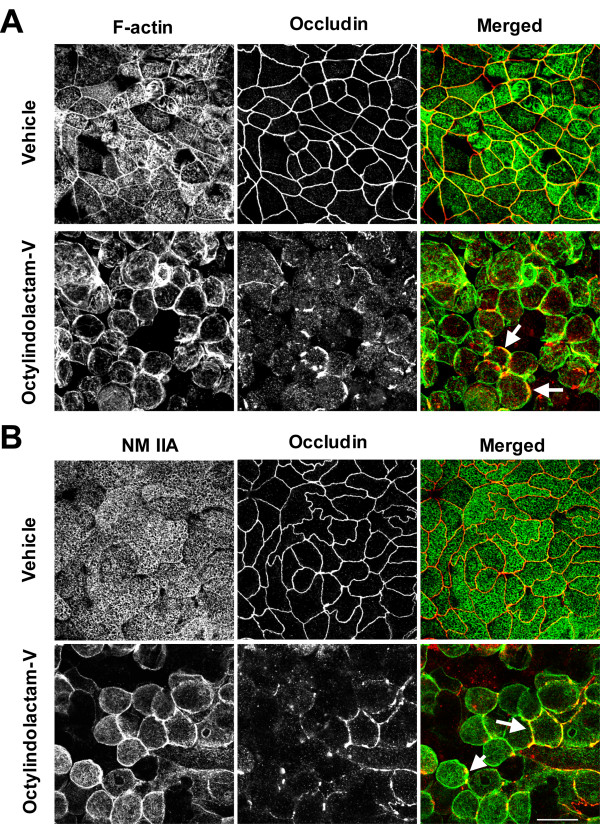
**Junctional disassembly in PKC-activated pancreatic epithelial cells is accompanied by reorganization of the apical actomyosin cytoskeleton**. Confluent HPAF-II cell monolayers were exposed for 3 h to either vehicle or OI-V (1 μM) followed by fixation and dual fluorescence labeling for occludin (red) with either F-actin or NM IIA heavy chain (green). OI-V induces cell rounding and accumulation of F-actin and NM IIA at the lateral plasma membrane in the areas of disassembling TJ (arrows). Bar, 20 μm.

Given the critical role of the actomyosin cytoskeleton in regulation of epithelial junctions, we next sought to investigate its involvement in PKC-induced disassembly of TJs and AJs in pancreatic epithelium. In control HPAF-II monolayers, staining with fluorescently-labeled phalloidin revealed a prominent F-actin belt at the level of apical junctions along with fine punctate apical staining representing microvilli F-actin (Figure 4A). PKC activation induced cell rounding associated with the lateral condensation of actin filaments (Figure 4A, arrows), which are suggestive of increased F-actin contractility in these areas [33,40]. To further characterize these putative contractile events, we analyzed effects of OI-V on localization of the F-actin motor, NM II. Epithelial cells express three different NM II heavy chain isoforms, IIA, IIB, and IIC [40,61], and NM IIA is the most abundant isoform in HPAF-II cells (data not shown). In control HPAF-II monolayers, immunofluorescence analysis revealed predominant localization of NM IIA at the apical surface (Figure 4B). Upon PKC activation, NM IIA translocated to the lateral plasma membrane in the areas of F-actin condensation and AJ/TJ disassembly (Figure 4B, arrows).

**Figure 5:**
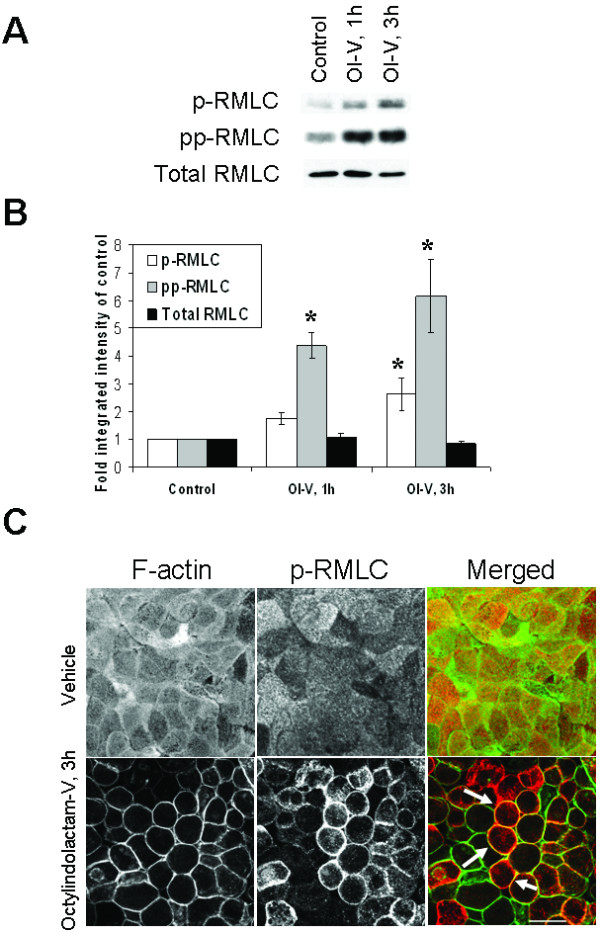
**PKC-activating tumor promoter induces rapid activation of myosin II**. (**A**) Representative Western blots and (**B**) densitometric quantification show an increase in the amounts of mono-phosphorylated (p) and di-phosphorylated (pp), but not total regulatory myosin light chain (RMLC) in HPAF-II lysates after 1 and 3 h exposure to OI-V (1 μM). Data are presented as mean ± SE (n = 4); *p < 0.05 compared to the vehicle-treated group. (**C**) Double-fluorescence labeling of p-RMLC (red) and F-actin (green) shows diffuse apical staining of p-RLMC in control HPAF-II cells and a lateral accumulation of p-RLMC (arrows) after 3 h of the OI-V treatment. Bar, 20 μm.

Since OI-V altered cellular localization of NM II, we next analyzed whether OI-V also affected its activation status. NM II activity is regulated by the phosphorylation of the regulatory myosin light chain (RMLC) on either one (Ser 19) or two (Thr18/Ser19) residues [62,63]. By using immunoblotting analysis, we compared levels of mono- and diphosphorylated RMLC in control and OI-V-treated HPAF-II cells and observed an early and dramatic increase in the amount of monophosphorylated (p) and diphosphorylated (pp) RMLC at 1–3 h after OI-V treatment (Figure 5A, B). Furthermore, PKC activation altered intracellular localization of activated NM II. Indeed, while in control HPAF-II cells p-RMLC was diffusely distributed within apical actin filaments, OI-V treated cells demonstrated significant accumulation of p-RMLC at lateral F-actin bundles localized in areas of disintegrating cell-cell contacts (Figure 5C, arrows).

**Figure 6:**
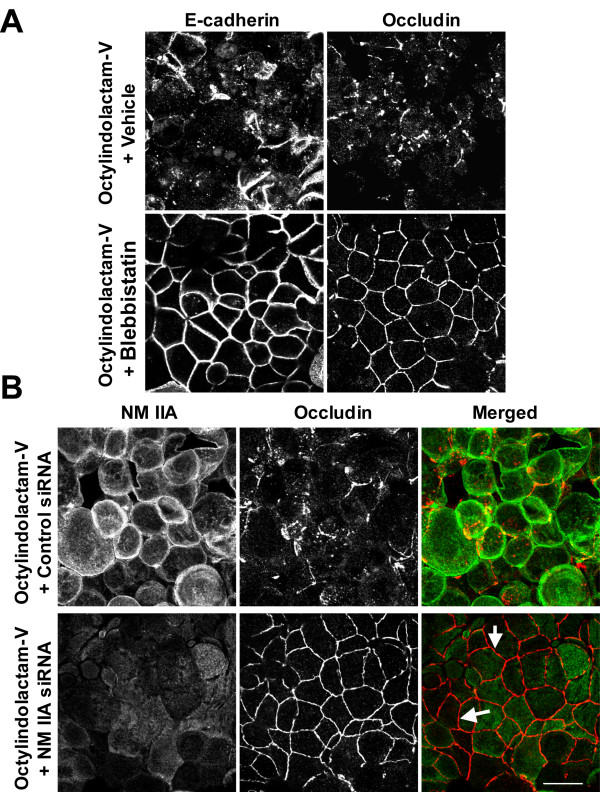
**Inhibition of NM II attenuates disassembly of epithelial apical junctions induced by activation of PKC**. (**A**) HPAF-II cells were treated for 5 h with either OI-V alone (1 μM), or in a combination with pharmacological inhibitors of NM II, blebbistatin (100 μM). Blebbistatin significantly attenuates disassembly of E-cadherin-based AJ and occludin-based TJ induced by the PKC activator. (**B**) HPAF-II cells were transfected with either control or NM IIA-specific siRNAs and exposed to 1 μM OI-V for 5 h on day 3 post-transfection. In contrast to control cell monolayers, NM IIA-depleted cells do not respond to PKC activator by junctional disassembly and retain their occludin in the areas of cell-cell contacts (arrows). Bar, 20 μm.

To obtain definitive evidence for the role of NM II in OI-V-induced junctional disassembly we inactivated NM II by using either the pharmacological inhibitor, blebbistatin [64] or siRNA-mediated depletion of NM IIA. Incubation with blebbistatin (100 μM) significantly attenuated disruption of TJs and AJs induced by 5 h exposure of HPAF-II cell monolayers to OI-V (Figure 6A). Furthermore, RNA interference, which caused ~68% decrease in NM IIA expression (Additional File 3), attenuated disassembly of apical junctions. Indeed, while control siRNA transfected HPAF-II cells lost the majority of their occludin-based TJs, cells with decreased NM IIA level retained morphologically-intact TJs after 5 h of OI-V treatment (Figure 6B, arrows). Interestingly, NM II inhibition while preserving TJ structure did not prevent PKC-dependent increase in paracellular permeability. Indeed, 3 h exposure of HPAF-II cells to OI-V resulted in TEER drop from 1555 ± 87 to 30 ± 2 Ohm × cm^2 ^(98%) and from 1046 ± 20 to 47 ± 3 Ohm × cm^2 ^(95%) in vehicle- and blebbistatin-treated cell monolayers respectively. Together these data suggest that NM II plays an important role in the disruption of AJ and TJ structure caused by PKC activation in pancreatic epithelium.

### Inhibition of Rho associated kinase, but not RhoA GTPase attenuated OI-V-induced disassembly of epithelial junctions

**Figure 7:**
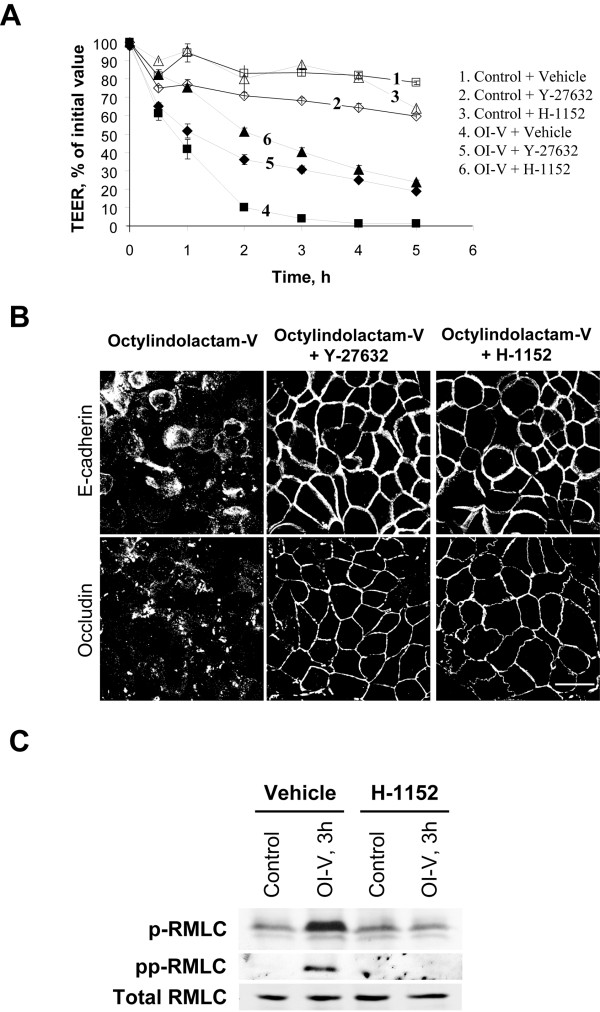
**Pharmacological inhibition of Rho-dependent kinase (ROCK) prevents OI-V-induced disruption of apical junctions**. HPAF-II cells were treated for 5 h with either OI-V alone (1 μM) or in a combination with two pharmacological ROCK inhibitors, Y-27632 (20 μM) and H-1152 (10 μM). The effects of the PKC activator on barrier properties were determined by TEER measurement (**A**), whereas the integrity of AJ and TJ was analyzed by immunofluorescence labeling for E-cadherin and occludin respectively (**B**). Both ROCK inhibitors significantly attenuate OI-V-induced disruption of paracellular barrier and disassembly of AJ and TJ. Bar, 20 μm. (**C**) Immunoblotting analysis shows that inhibition of ROCK using H-1152 (10 μM) abolishes both mono- and di-phosphorylation of RMLC induced by 3 h exposure to OI-V (1 μM), while not affecting the levels of total RMLC.

**Figure 8:**
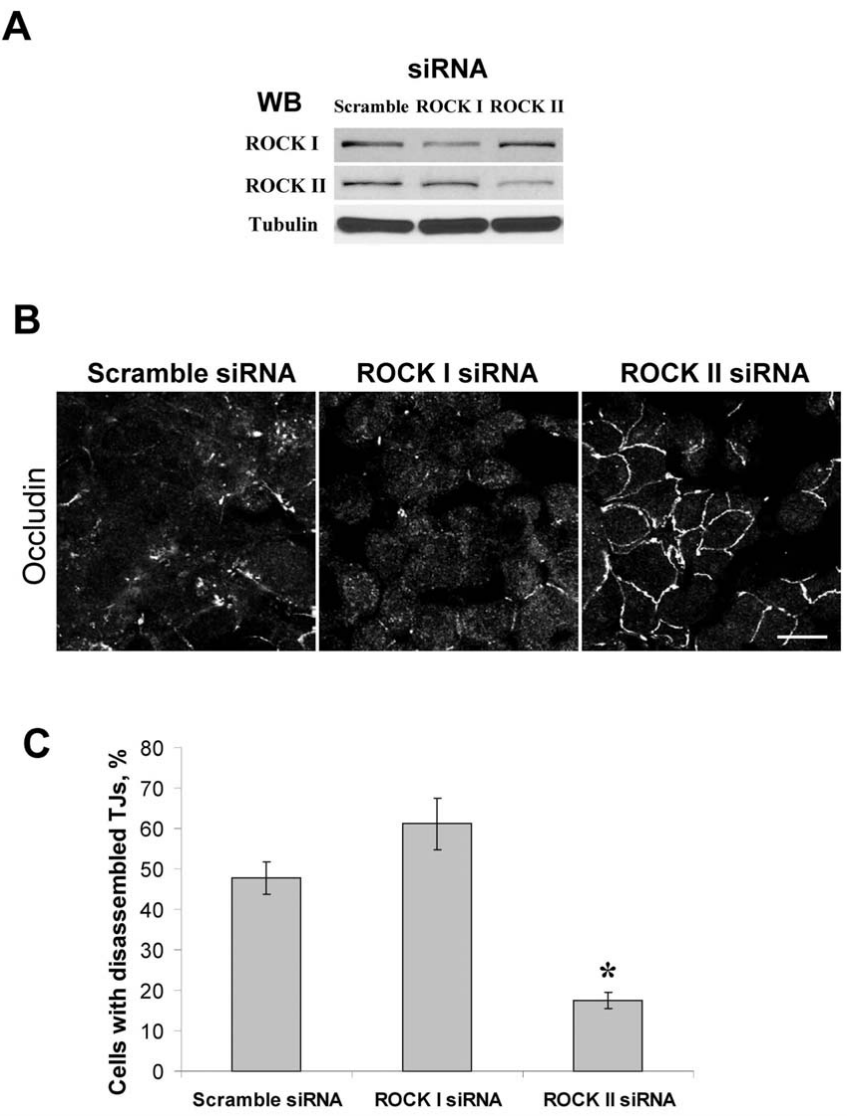
**siRNA-mediated knock-down of the ROCK-II isoform selectively attenuates PKC-dependent disassembly of TJ**. HPAF-II cells were transfected with either control or ROCK-isoform specific siRNAs and treated with OI-V (1 μM) for 5 h on day 3 post-transfection. (**A**) Immunoblotting analysis shows effective down-regulation of ROCK-I and ROCK-II by corresponding siRNAs. (**B**) Down-regulation of ROCK-II attenuates OI-V-induced TJ disassembly whereas ROCK-I knockdown has no effect. Bar, 20 μm. (**C**) Quantitative analysis of TJ disassembly. Data are presented as mean ± SE (n = 3); *p < 0.05 compared to the control siRNA-transfected cells.

Since PKC does not directly phosphorylate RMLC at Ser19 and Thr18 to stimulate actomyosin contractility [65,66] these events should be mediated by other protein kinases. The most likely candidates are Rho-associated kinase (ROCK) and myosin light chain kinase (MLCK), which have been previously implicated in the activation of perijunctional NM II and AJ/TJ disassembly triggered by various stimuli [42,43,67,68]. To analyze the involvement of ROCK in the OI-V-induced disruption of apical junctions we used two structurally unrelated pharmacological ROCK inhibitors, Y-27632 (20 μM) and H-1152 (10 μM). Both compounds significantly attenuated OI-V-induced drop in TEER (Figure 7A) and inhibited disassembly of E-cadherin-based AJs and occludin-based TJs (Figure 7B). In agreement with these functional data H-1152 also suppressed OI-V-dependent increase in RLMC phosphorylation (Figure 7C). We next used RNA interference to confirm these pharmacological inhibition results and to identify which ROCK isoform, ROCK-I or ROCK-II, is involved. ROCK-I and ROCK-II expression was selectively downregulated by ~65% and ~70% respectively in HPAF-II cells using isoform-specific siRNA duplexes (Figure 8A). siRNA-mediated depletion of ROCK-II, but not ROCK-I significantly attenuated OI-V-induced disassembly of epithelial TJs (Figure 8B,C) and AJs (data not shown).

**Figure 9:**
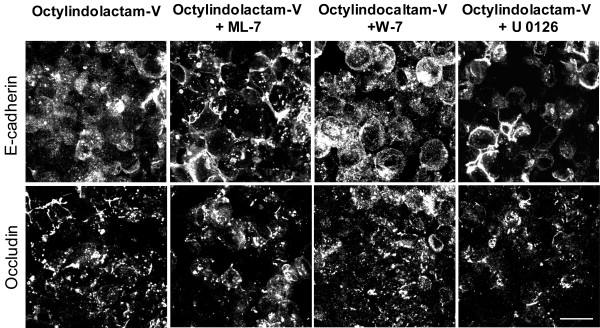
**Inhibition of MLCK, calmodulin, and ERK1/2 does not affect OI-V-induced disassembly of epithelial apical junctions**. HPAF-II cells were treated for 5 h with either OI-V alone (1 μM) or in a combination with pharmacological inhibitors of MLCK (ML-7 (20 μM)), calmodulin (W-7 (100 μM)), and ERK1/2 (U 1026 (5 μM)). The integrity of AJ and TJ was determined by immunofluorescence labeling for E-cadherin and occludin respectively. Neither inhibitor prevents OI-V-induced disassembly of AJ and TJ. Bar, 20 μm.

To elucidate whether ROCK II plays a unique role in PKC-dependent disruption of epithelial junctions, we next tested the involvement of alternative signaling pathways previously implicated in either NM II activation, or PKC-dependent disassembly of cell-cell adhesions [29,42,43,69]. We used pharmacological inhibitors, ML-7 (20 μM), W-7 (100 μM) and U 0126 (10 μM), which block the activity of MLCK, calmodulin (a cofactor of MLCK), and extracellular signal regulated kinases (ERK) 1/2 respectively, and found that neither inhibitor affected OI-V-induced junctional disassembly in HPAF-II cells (Figure 9). These data highlight a unique role for ROCK II in the signaling cascade, which is initiated by PKC activation and leads to junctional breakdown.

**Figure 10:**
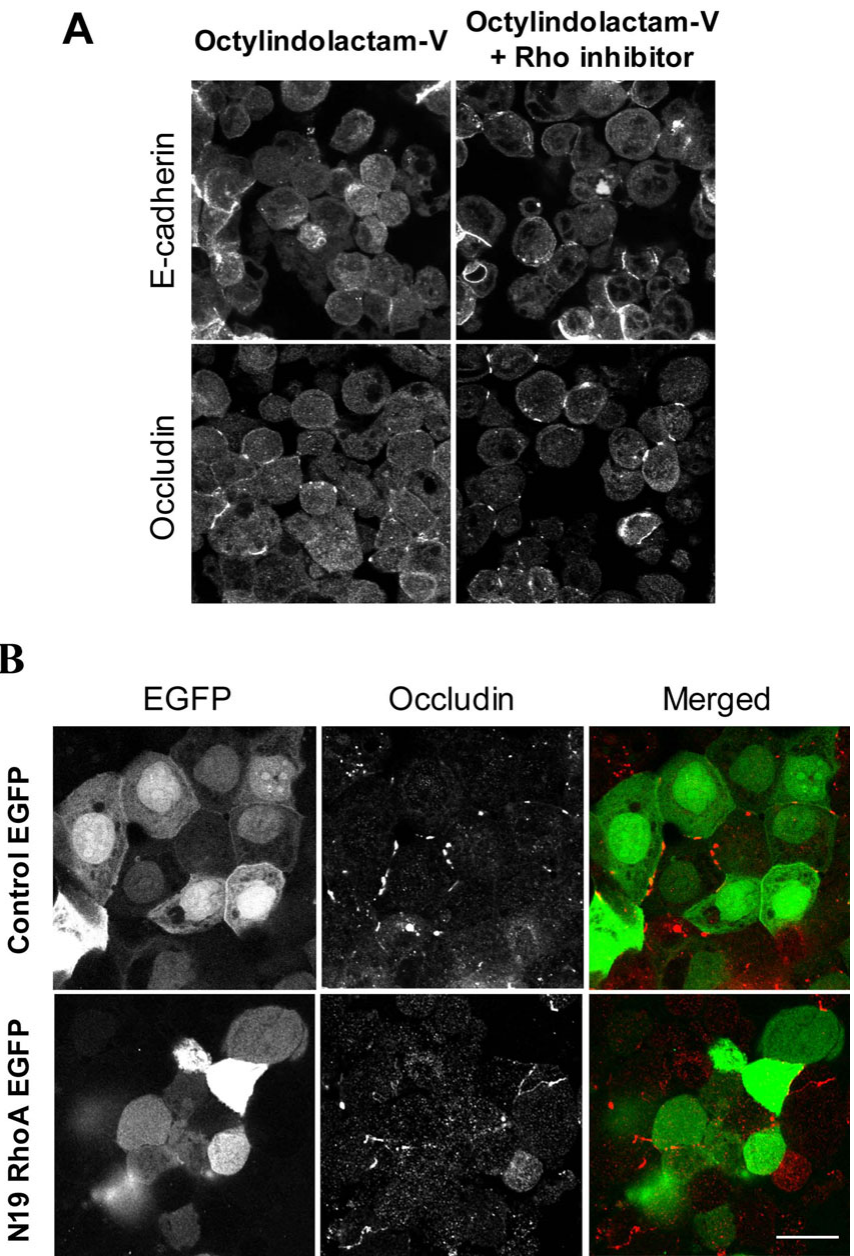
**Inhibition of RhoA GTPase does not prevent OI-V-induced disassembly of epithelial apical junctions**. Activity of Rho GTPase in HPAF-II cells was blocked by either incubation with a cell-permeable inhibitor C3 toxin (2 μg/ml for 3 h; **A**) or overexpression of a dominant-negative N19 RhoA mutant (**B**), and effect of this inhibition on OI-V induced disassembly of AJ and TJ was determined by immunofluorescence labeling and confocal microscopy. Neither pharmacological inhibitor, nor dominant-negative RhoA mutant prevent disassembly of epithelial junctions induced by the PKC activator. Bar, 20 μm.

Since ROCK represents the major downstream effector for Rho small GTPase [70,71], we investigated whether RhoA mediates OI-V-induced disassembly of epithelial junctions. Two different approaches were used to inhibit RhoA activity in HPAF-II cells. One involved pharmacological inhibition of Rho with cell-permeable *Clostridium botulinum *C3 exoenzyme [72] and the other employed the overexpression of a dominant-negative (N19) RhoA mutant. We observed that pharmacological Rho inhibitor failed to prevent OI-V-induced disassembly of TJs (Figure 10A) and an increase in RMLC phosphorylation (Additional File 4A). It is noteworthy, that this inhibitor caused disruption of basal F-actin bundles in confluent HPAF-II monolayers (Additional File 4B), which indicates its activity in our experimental conditions. Likewise, the dominant-negative RhoA mutant had no effect on junctional disassembly in OI-V treated cell monolayers (Figure 10B). Finally, we sought to investigate if tumor promoter-depended disruption of AJs and TJs can be initiated by pro-apoptotic signaling. Apoptosis in control and OI-V-exposed HPAF-II cell monolayers was examined by using fluorescent pan-caspase activation detection kit. As shown in Additional File 5A, OI-V treatment did not result in significant caspase activation thus indicating lack of apoptosis induction. Furthermore, pretreatment with a pan-caspase inhibitor z-VAD-fmk (50 μM) failed to prevent OI-V-induced AJ disassembly (Additional File 5B). Overall, these results suggest that mechanism of OI-V-induced AJ/TJ disassembly in pancreatic epithelial cells involves activation of actomyosin contractility via RhoA- and apoptosis-independent ROCK-II signaling.

## Discussion

Loss of epithelial cell-cell adhesions upon exposure to PKC-activating carcinogens is commonly used to model scattering of epithelial cells during tumor metastasis [19,22,25]. In this study, we investigate molecular mechanisms underlying tumor promoter-induced disassembly of epithelial apical junctions by challenging HPAF-II human pancreatic epithelial cell monolayers with OI-V or TPA. HPAF-II cells have been derived from highly metastatic pancreatic adenocarcinoma. These cells polarize in culture and develop a high resistance paracellular barrier with well-defined TJs and AJs. In addition, these cells are amenable to transfection with siRNAs. Together, these features make HPAF-II cells an excellent *in vitro *model to study disruption of epithelial cell-cell adhesions during tumor progression/metastasis.

Exposure of HPAF-II cell monolayers to OI-V or TPA induced rapid opening of the paracellular barrier (Figure 1) and disassembly of AJs and TJs (Figure 2). Since both agents are known to activate PKC, it is logical to suggest that such a junctional disassembly is triggered by activation of PKC. Our pharmacological inhibition and biochemical analyses confirmed the role of PKC activation in disruption of epithelial AJs and TJs and indicated that classical PKC isoenzymes are responsible for this biological effect (Figure 3 and Additional File 2). Different classes of PKC have been previously implicated in phorbol ester-mediated opening of epithelial barriers. Our data are consistent with results of two studies that have implicated classical PKC in TPA-induced increase in paracellular permeability in LLC-PK1 [28] and T84 cell monolayers [73]. However, some other studies have suggested the role of novel PKC isoforms in TPA-induced internalization of AJ protein E-cadherin in MDCK cells [20] and disruption of claudin-4-based TJ in OVCA433 ovarian epithelial cells [74]. Since TPA and OI-V activate both classical and novel PKC, distinct roles of these subfamilies in AJ/TJ disassembly in different epithelia is likely to reflect cell-specific differences in their expressional levels and/or junctional association. It is noteworthy, that pharmacological inhibitors do not allow identification of the individual PKC isoform, which is responsible for AJ/TJ disruption in HPAF-II cell monolayers. Further studies involving more selective RNA interference or dominant-negative PKC mutant approaches are required to answer this important question.

A key finding of this study is a critical role of NM II in disassembly of AJs and TJs upon PKC activation in HPAF-II epithelial cells. It should be noted that the involvement of actomyosin contractility in PKC-dependent disruption of cell-cell adhesions has been addressed in previous publication, which yielded conflicting results. Thus, the increased F-actin tension/contraction has been implicated in phorbol-ester-induced disruption of endothelial junctions in one [66], but not another [75] study. Furthermore, TPA was shown to either increase [76,77], have no effect [66] or decrease [47,75,78] RMLC phosphorylation in endothelial and epithelial cell monolayers. Our conclusion that PKC activation disrupts epithelial AJs and TJs via stimulating NM II activity is based on several lines of evidence. First, OI-V triggered NM II activation in HPAF-II cells at the onset of junctional disassembly (Figure 5). Second, NM II relocalized to disassembling junctions in parallel with cell rounding in OI-V-treated cells (Figure 4B, 5C). Finally, inhibition of NM II significantly attenuated OI-V-induced disassembly of AJs and TJs (Figure 6).

Our study provides the first direct evidence implicating NM II activity in the disruption of epithelial apical junctions by PKC-activating tumor promoters. Such activation of NM II is likely to serve as a trigger for junctional disassembly by either breaking adhesive contacts formed by transmembrane junctional proteins, or by destabilizing perijunctional F-actin bundles. This may activate endocytosis of AJ/TJ proteins, thus leading to complete disintegration of apical junctions [19,25]. Although the role of NM II in the breakdown of epithelial barrier in inflammation is generally accepted [79,80], it has not been explored whether similar mechanism mediates disruption of epithelial cell-cell adhesions in tumorigenesis. Based on the present *in vitro *data, we hypothesize that stimulation of actomyosin contractility can also be involved in the loss of epithelial cell-cell contacts during metastatic scattering of tumor cells *in vivo*.

Although PKC was shown to directly phosphorylate RMLC at Ser1/2 residues [65,66], such phosphorylation cannot be responsible for the increased level of Ser19/Thr18-phosphorylated RLMC and stimulation of actomyosin contractility. In a search for an intermediate signaling step, which links PKC activation and stimulation of NM II, we identified ROCK as a critical regulator of junctional disassembly in OI-V-challenged HPAF-II cells (Figures 7 &8). Importantly, selective siRNA-mediated knock-down of ROCK-I and ROCK-II showed a unique role of the latter isoform in the PKC-dependent disruption of epithelial junctions (Figure 8). These results are in a good agreement with our recent study, which demonstrated the involvement of ROCK-II, but not ROCK-I in AJ/TJ disassembly induced by the depletion of extracellular calcium in intestinal epithelial cells [68]. Although ROCK-I and ROCK-II are highly homologous (~65% of sequence identity and 92% identity in their kinase domain) [81], these isoforms can be differentially regulated, and can activate distinct actomyosin-dependent processes [82,83]. For example, ROCK-II, but not ROCK-I, binds to inositol phospholipids, and is activated by phosphoinositol-3-kinase [82]. These different regulatory mechanisms are likely to underline a selective involvement of ROCK-II in the dynamics of epithelial apical junctions. Importantly, our pharmacological analysis failed to observe the involvement of MLCK or ERK-mediated signaling in PKC-dependent AJ/TJ disassembly (Figure 9), which further supports a unique role of ROCK in stimulating NM II-dependent contractility that disrupts epithelial junctions.

Inhibition of NM II and ROCK while substantially attenuating OI-V-induced disintegration of AJ/TJ structure either did not affect, or only modestly attenuated the decrease in TEER (Figures 6 &7). This suggests that PKC activation compromises integrity of the epithelial barrier via several pathways including, but not limited to ROCK and NM II. Additional mechanisms may involve PKC-dependent changes in phosphorylation of different junctional components, which perturbs normal protein-protein interactions within the AJ/TJ complexes. Indeed, altered phosphorylation of occludin and p120 catenin was observed in epithelial and endothelial cells exposed to PKC-activating tumor promoters [84]

One intriguing findings of this study is that the signaling cascade, which is triggered by PKC activation and mediated by ROCK-II does not involve RhoA GTPase (Figure 10). This implies that PKC can bypass RhoA in stimulating ROCK-II activity and is consistent with a recent report demonstrating PKC-dependent ROCK activation downstream of RhoA in human endothelial cells [85]. Our findings also agree with data obtained in other experimental systems which demonstrated that RhoA inhibition did not prevent TPA-induced contraction in cerebrovascular smooth muscle [86] or TPA-mediated reorganization of NM II in CHO cells [87]. Lack of involvement of RhoA in OI-V-induced junctional disassembly indicates that this process may be either regulated by other Rho isoforms such as RhoC and RhoE, or may involve Rho-independent mechanisms.

Recent studies have unraveled a complexity of mechanisms regulating ROCK activity which also involves Rho-independent activatory modes. For example, a Rho-independent cleavage of ROCK, that renders this enzyme constitutively active, was reported by several groups [88-91]. Particularly, ROCK-II was shown to be cleaved by caspase-2 in thrombin-stimulated endothelial cells [89] or by granzyme B in apoptotic lymphocytes [90]. However, this mechanism is not responsible for ROCK-II activation by PKC-targeting tumor promoters in model pancreatic epithelium, since OI-V did no induce ROCK-II cleavage in HPAF-II cells (data not shown), and caused junctional disassembly in caspase-independent fashion (Additional File 5). Further studies are required to elucidate mechanisms of PKC-dependent activation of ROCK-II in epithelial cells.

## Conclusion

This study dissected critical intracellular events which are involved in disassembly of epithelial apical junctions stimulated by the tumor promoter, octylindolactam V. These events involve activation of classical PKC isoforms, which bypasses RhoA and signals directly through ROCK-II to activate NM II. Activation of NM II stimulates reorganization/contractility of perijunctional actomyosin ring, which drives the disassembly of epithelial AJs and TJs. Similar mechanisms may contribute to the disruption of cell-cell adhesions during tumor progression and metastasis.

## Methods

### Antibodies and other reagents

The following primary polyclonal (pAb) and monoclonal (mAb) antibodies were used to detect junctional, cytoskeletal and signaling proteins: anti-occludin, ZO-1, E-cadherin, and β-catenin mAbs (Invitrogen, Carlsbad, CA); anti-NM IIA pAb (Covance, Berkley, CA); anti-PKCα mAb, anti-PKC βI, anti-ROCK-I (H-85), anti-ROCK-II (C-20 and H-85), and anti-RMLC pAbs (Santa Cruz Biotechnology, Santa Cruz, CA); anti-PKCδ pAb (Millipore, Billerica, MA) anti-mono, and di-phosphorylated RMLC pAbs (Cell Signaling Technology Inc., Beverly, MA); anti-actin pAb (Sigma-Aldrich, St. Louis, MO). Alexa-488 or Alexa-568 dye conjugated donkey anti-rabbit and goat anti-mouse secondary antibodies and Alexa-labeled phalloidin were obtained from Molecular Probes (Eugene, OR); horseradish peroxidase-conjugated goat anti-rabbit and anti-mouse secondary antibodies were obtained from Jackson Immunoresearch Laboratories (West Grove, PA). (-)-7-Octylindolactam V was obtained from Biomol International (Plymouth Meeting, PA); TPA, S(-)-Blebbistatin, W-7, ML-7 and rottlerin were purchased from Sigma; Gö 6976, Y-27632, H-1152, and U0126 were purchased from EMD Biosciences (San Diego, CA); GF-109203X was obtained from Axxora LLC (San Diego, CA); a cell permeable Rho inhibitor was obtain from Cytoskeleton Inc. (Denver, CO). All other reagents were of the highest analytical grade and obtained from Sigma.

### Cell culture and pharmacological modulation of junctional disassembly

HPAF-II human pancreatic epithelial cells (American Type Culture Collection, Manassas, VA) were grown in RPMI medium supplemented with 10% fetal bovine serum, 10 mM HEPES, 1 mM sodium pyruvate, 2 mM L-glutamine, 100 IU/ml penicillin and 100 μg/ml streptomycin, pH 7.4. For immunofluorescence labeling experiments epithelial cells were grown for 6–10 days on either collagen-coated permeable polycarbonate Transwell filters with 0.4 μm pore size (Costar, Cambridge, MA) or on collagen-coated coverslips. For biochemical experiments cells were cultured on either Transwell filters, or 6-well plastic plates. Junctional disassembly in HPAF-II cell monolayers was induced by incubation with either OI-V or TPA for indicated periods of time. For pharmacological inhibition experiments, cells were preincubated with inhibitors for 30 min followed by exposure to OI-V in the presence of inhibitors. Appropriate vehicle (DMSO) was added to all control samples.

### Immunofluorescence labeling and image analysis

Cell monolayers were fixed/permeabilized in 100% methanol or 100% ethanol (-20°C for 20 min) and double-immunolabeled according to previously described protocols [33,35,40]. For visualization of F-actin cells were fixed in 3.7% paraformaldehyde for 15 min and subsequently permeabilized with 0.5% Triton X-100 for 10 min at room temperature. Stained cells were examined using Zeiss LSM510 laser scanning confocal microscope (Zeiss Microimaging Inc., Thornwood, NY) equipped with 63× and 100× Pan-Apochromat oil lenses. The Alexa Fluor 488 and 555 signals were imaged sequentially in frame-interlace mode to eliminate cross-talk between channels. Images were processed using Zeiss LSM5 image browser software and their brightness and contrast were adjusted in Adobe Photoshop. Images shown are representative of at least 3 independent experiments with multiple images taken per slide. The statistical analysis of TJ disassembly was performed as described previously [68]. Briefly, cells were co-stained for occludin (or E-cadherin) and nuclei, and at least 10 random images per slide were acquired at the level of TJ using 63× objective. The total number of cells per image was determined by nuclei count. The cells with diffuse cytoplasmic localization of occludin, which showed no discontinuous staining pattern with the other cells, were counted as cells with disassembled TJ. If neighbor cells showed common discontinuous occludin localization pattern, those cells were counted as cells maintaining TJ.

### Immunoblotting

Cells were homogenized in a RIPA lysis buffer (20 mM Tris, 50 mM NaCl, 2 mM EDTA, 2 mM EGTA, 1% sodium deoxycholate, 1% TX-100, and 0.1% SDS, pH 7.4), containing a protease inhibitor cocktail (1:100, Sigma) and phosphatase inhibitor cocktails 1 and 2 (both at 1:200, Sigma). Lysates were cleared by centrifugation (10 min at 14,000 × g), diluted with 2× SDS sample buffer and boiled. SDS-polyacrylamide gel electrophoresis and immunoblotting were conducted by standard protocols with equal amount of total protein (10 or 20 μg) per lane. Results shown are representative immunoblots of three independent experiments. Protein expression was quantified by densitometric analysis of at least three Western blot images each representing independent experiment, using Scion Image (Scion, Frederick, MD) and UN-SCAN-IT digitizing software (Silk Scientific, Orem, UT).

### Cell fractionation

Cytosolic and membrane fractions of control or OI-V treated HPAF-II cells were prepared as described previously [92]. Briefly, cells were harvested in Relax buffer (100 mM KCl, 3 mM NaCl, 3.5 mM MgCl_2_, 10 mM Hepes, pH 7.4) containing protease and phosphatase inhibitor cocktails and nitrogen cavitated (200 psi, 15 min). Nuclei were removed by centrifugation (1,000 × g × 10 min). Membranes were pelleted by ultracentrifugation of the post nuclear supernatants at 100,000 × g for 60 min. Supernatants (cytosolic fraction) were collected and the pellets were resuspended in an equivalent volume (equal to the starting volume of cell lysate) of Hanks balanced salt solution containing 1% n-octylglucoside by sonication on ice. Equal volumes of the cytosolic and membrane fractions were subjected to immunoblotting analysis as described above.

### RT-PCR

Total RNA was isolated from confluent HPAF-II cells using Trizol LS extraction reagent (BD Biosciences, Franklin Lakes, NJ) according to manufacturer's protocol followed by DNase I digestion (Promega, Madison, WI). RT-PCR reaction was performed using a SuperScript III One-Step RT-PCR System (Invitrogen). The primer sequences of different PKC isoforms are presented in the Additional File 1B. Specificity of amplification was verified by running agarose electrophoresis of each amplicon and obtaining a single band of expected size. To control for genomic contamination and other nonspecific products, SuperScript III RT in PCR reaction was substituted with Taq DNA polymerase (a negative control line in the Additional File 1A).

### RNA interference and adenoviral infection

siRNA-mediated knock-down was carried out using isoform-specific human NM IIA siRNA SmartPool (Dharmacon, Lafayette, CO), p160ROCK (ROCK-I) duplex 2 and ROCK-II duplex 1 (QIAGEN, Valencia, CA) respectively. Cyclophilin B siRNA SmartPool (Dharmacon) was used as a control. HPAF-II cells were transfected using the DharmaFect 1 transfection reagent (Dharmacon) in Opti-MEM I medium (Invitrogen) according to manufacturer's protocol with a final siRNA concentration of 50 nM. Cells were used in experiments 72–80 h post-transfection. Adenoviruses expressing EGFP-tagged dominant negative N19RhoA mutant, as well as control EGFP virus were provided by Dr. James Bamburg (Colorado State University) and produced as described previously [93]. Cells were incubated in DMEM medium containing purified viral particles diluted to 5 × 10^5 ^plaque-forming units/ml for 36 h before OI-V treatment.

### Transepithelial electrical resistance measurement

Effect of PKC activators on transepithelial electrical resistance was measured using an EVOMX voltohmmeter (World Precision Instruments, Sarasota, FL). The resistance of cell-free collagen-coated filters was subtracted from each experimental point.

### Caspase activation assay

Confluent HPAF-II monolayers were incubated for 5 h with either vehicle, octylindolactam-V (1 μM), or camptothecin (2 μg/mL). Live cells were labeled with Poly Caspase FLICA detection Kit (Axxora LLC) according to the manufacturer protocol, fixed in 3.7% paraformaldehyde and examined by confocal microscopy as described above.

### Statistics

Numerical values from individual experiments were pooled and expressed as mean ± standard error of the mean (S.E.) throughout. The results were compared by a post-hoc Bonferroni test following analysis of variance (ANOVA) with a statistical significance assumed at p < 0.05.

## List of Abbreviations

AJs: adherens junctions; EMT: epithelial to mesenchymal transition; OI-V: octylindolactam V; NM II: nonmuscle myosin II; PKC: protein kinase C; ROCK: Rho-dependent kinase; TEER: transepithelial electrical resistance; TJs: tight junctions; TPA: 12-O-tetradecanoylphorbol-13-acetate; ZO-1: 'zonula occludens'-1.

## Authors' contributions

AII conceived of the study, carried out siRNA-mediated gene knockdown, inhibitory analysis, immunofluorescence labeling and drafted the manuscript. SNS participated in confocal microscopy data acquisition, permeability measurements and siRNA knockdowns. MB carried out Western blot analysis. CAP and AN participated in design and coordination of the study and helped to draft the manuscript.

## Supplementary Material

Additional file 1 **Detection of different PKC isoforms in HPAF-II cells**. The data provided represent the RT-PCR analysis of different PKC isoform expression in confluent HPAF-II cell monolayers. (A) Agarose electrophoresis of PCR amplicons shows expression of two classical (PKCs α and γ) and four novel (PKCs δ, ε, η and θ) PKC isoforms. (B) Primer sequences that were used to detect expression of different PKC isoenzymes. Click here for file

Additional file 2 **OI-V induces membrane translocation of different PKC isoforms**. The data presented show distribution of different PKC isoenzymes between cytosolic and plasma membrane fractions in control and OI-V-treated epithelial cells. Note that 1 h OI-V treatment rapidly increased the amount of both classical (α and βI) and novel (δ) PKC isoenzymes associated with cell membranes. Click here for file

Additional file 3 **NM IIA-specific siRNAs effectively downregulate expression of this protein**. The data show the effectiveness of siRNA-mediated depletion of NM IIA. HPAF-II cells were transfected with either a control (cyclophilin B), or NM IIA-specific siRNA SmartPools and analyzed for NM IIA expression 84 h post-transfection. Click here for file

Additional file 4 **Effects of Rho inhibition on myosin phosphorylation and basal F-actin filaments in OI-V-treated and control epithelial cells**. The presented data demonstrate different effects of Rho inhibition on actomyosin cytoskeleton in HPAF-II cells. (A) HPAF-II cells were treated for 3 h with either OI-V alone (1 μM), or in a combination with cell-permeable Rho inhibitor, C3 toxin (2 μg/ml). Immunoblotting analysis shows that Rho inhibitor fails to prevent OI-V-dependent increase in the amount of mono- and di-phosphorylated RMLC. (B) Control HPAF-II monolayers were treated for 3 h with either vehicle or C3 toxin with subsequent fixation and fluorescence labeling of F-actin. Note that Rho inhibitor causes disappearance of basal actin filaments in HPAF-II cell monolayers which indicates the efficiency of this inhibitor in our experimental conditions. Bar, 20 μm. Click here for file

Additional file 5 **OI-V-induced junctional disassembly is independent of apoptosis**. These experiments probed the role of caspase activation in OI-V-induced junctional disassembly (A) Confluent HPAF-II cell monolayers were incubated for 5 h with either vehicle, or OI-V. Cell monolayers exposed for 5 h to a classical pro-apoptotic agent, camptothecin (2 μg/mL), were used as a positive control. Cell monolayers were fixed and probed with Poly Caspase FLICA detection kit. Note, that camptothecin-treated cells show a significant increase in the number of FLICA-positive caspase-activated cells (green), whereas OI-V does not induce such a caspase activation. (B) HPAF-II cell monolayers were treated for 3 h with either vehicle or 1 μM OI-V with or without pretreatment with a potent pan-caspase inhibitor zVAD-fmk (50 μM). Junctional integrity was evaluated by immunolabeling for E-cadherin. Note that caspase inhibition has no effect on AJ disassembly induced by OI-V. Bar, 20 μm. Click here for file

## References

[B1] Guarino M, Rubino B, Ballabio G (2007). The role of epithelial-mesenchymal transition in cancer pathology. Pathology.

[B2] Thiery JP, Sleeman JP (2006). Complex networks orchestrate epithelial-mesenchymal transitions. Nat Rev Mol Cell Biol.

[B3] Guarino M (2007). Epithelial-mesenchymal transition and tumour invasion. Int J Biochem Cell Biol.

[B4] Savagner P (2001). Leaving the neighborhood: molecular mechanisms involved during epithelial-mesenchymal transition. Bioessays.

[B5] Hartsock A, Nelson WJ (2008). Adherens and tight junctions: Structure, function and connections to the actin cytoskeleton. Biochim Biophys Acta.

[B6] Pokutta S, Weis WI (2007). Structure and mechanism of cadherins and catenins in cell-cell contacts. Annu Rev Cell Dev Biol.

[B7] Tsukita S, Furuse M, Itoh M (2001). Multifunctional strands in tight junctions. Nat Rev Mol Cell Biol.

[B8] Miyoshi J, Takai Y (2008). Structural and functional associations of apical junctions with cytoskeleton. Biochim Biophys Acta.

[B9] Matter K, Balda MS (2007). Epithelial tight junctions, gene expression and nucleo-junctional interplay. J Cell Sci.

[B10] Perez-Moreno M, Fuchs E (2006). Catenins: keeping cells from getting their signals crossed. Dev Cell.

[B11] Boyer B, Tucker GC, Valles AM, Franke WW, Thiery JP (1989). Rearrangements of desmosomal and cytoskeletal proteins during the transition from epithelial to fibroblastoid organization in cultured rat bladder carcinoma cells. J Cell Biol.

[B12] Friedl P (2004). Prespecification and plasticity: shifting mechanisms of cell migration. Curr Opin Cell Biol.

[B13] Larue L, Bellacosa A (2005). Epithelial-mesenchymal transition in development and cancer: role of phosphatidylinositol 3' kinase/AKT pathways. Oncogene.

[B14] Boyer B, Valles AM, Edme N (2000). Induction and regulation of epithelial-mesenchymal transitions. Biochem Pharmacol.

[B15] Castagna M (1987). Phorbol esters as signal transducers and tumor promoters. Biol Cell.

[B16] Irie K, Nakagawa Y, Ohigashi H (2004). Indolactam and benzolactam compounds as new medicinal leads with binding selectivity for C1 domains of protein kinase C isozymes. Curr Pharm Des.

[B17] Griner EM, Kazanietz MG (2007). Protein kinase C and other diacylglycerol effectors in cancer. Nat Rev Cancer.

[B18] Koivunen J, Aaltonen V, Peltonen J (2006). Protein kinase C (PKC) family in cancer progression. Cancer Lett.

[B19] Kamei T, Matozaki T, Sakisaka T, Kodama A, Yokoyama S, Peng YF, Nakano K, Takaishi K, Takai Y (1999). Coendocytosis of cadherin and c-Met coupled to disruption of cell-cell adhesion in MDCK cells – regulation by Rho, Rac and Rab small G proteins. Oncogene.

[B20] Le TL, Joseph SR, Yap AS, Stow JL (2002). Protein kinase C regulates endocytosis and recycling of E-cadherin. Am J Physiol Cell Physiol.

[B21] Ojakian GK (1981). Tumor promoter-induced changes in the permeability of epithelial cell tight junctions. Cell.

[B22] Jansen LA, Mesnil M, Jongen WM (1996). Inhibition of gap junctional intercellular communication and delocalization of the cell adhesion molecule E-cadherin by tumor promoters. Carcinogenesis.

[B23] Krendel M, Gloushankova NA, Bonder EM, Feder HH, Vasiliev JM, Gelfand IM (1999). Myosin-dependent contractile activity of the actin cytoskeleton modulates the spatial organization of cell-cell contacts in cultured epitheliocytes. Proc Natl Acad Sci USA.

[B24] Farshori P, Kachar B (1999). Redistribution and phosphorylation of occludin during opening and resealing of tight junctions in cultured epithelial cells. J Membr Biol.

[B25] Kanda I, Nishimura N, Nakatsuji H, Yamamura R, Nakanishi H, Sasaki T (2008). Involvement of Rab13 and JRAB/MICAL-L2 in epithelial cell scattering. Oncogene.

[B26] Mullin JM, Marano CW, Laughlin KV, Nuciglio M, Stevenson BR, Soler P (1997). Different size limitations for increased transepithelial paracellular solute flux across phorbol ester and tumor necrosis factor-treated epithelial cell sheets. J Cell Physiol.

[B27] Mullin JM, McGinn MT, Snock KV, Imaizumi S (1990). The effects of teleocidin and aplysiatoxin tumor promoters on epithelial tight junctions and transepithelial permeability: comparison to phorbol esters. Carcinogenesis.

[B28] Rosson D, O'Brien TG, Kampherstein JA, Szallasi Z, Bogi K, Blumberg PM, Mullin JM (1997). Protein kinase C-alpha activity modulates transepithelial permeability and cell junctions in the LLC-PK1 epithelial cell line. J Biol Chem.

[B29] Wang Y, Zhang J, Yi XJ, Yu FS (2004). Activation of ERK1/2 MAP kinase pathway induces tight junction disruption in human corneal epithelial cells. Exp Eye Res.

[B30] Le TL, Yap AS, Stow JL (1999). Recycling of E-cadherin: a potential mechanism for regulating cadherin dynamics. J Cell Biol.

[B31] Matsuda M, Kubo A, Furuse M, Tsukita S (2004). A peculiar internalization of claudins, tight junction-specific adhesion molecules, during the intercellular movement of epithelial cells. J Cell Sci.

[B32] Troyanovsky RB, Sokolov EP, Troyanovsky SM (2006). Endocytosis of cadherin from intracellular junctions is the driving force for cadherin adhesive dimer disassembly. Mol Biol Cell.

[B33] Ivanov AI, McCall IC, Parkos CA, Nusrat A (2004). Role for actin filament turnover and a myosin II motor in cytoskeleton-driven disassembly of the epithelial apical junctional complex. Mol Biol Cell.

[B34] Shen L, Turner JR (2005). Actin depolymerization disrupts tight junctions via caveolae-mediated endocytosis. Mol Biol Cell.

[B35] Utech M, Ivanov AI, Samarin SN, Bruewer M, Turner JR, Mrsny RJ, Parkos CA, Nusrat A (2005). Mechanism of IFN-gamma-induced endocytosis of tight junction proteins: myosin II-dependent vacuolarization of the apical plasma membrane. Mol Biol Cell.

[B36] Larsson C (2006). Protein kinase C and the regulation of the actin cytoskeleton. Cell Signal.

[B37] De La Cruz EM, Ostap EM (2004). Relating biochemistry and function in the myosin superfamily. Curr Opin Cell Biol.

[B38] Maciver SK (1996). Myosin II function in non-muscle cells. Bioessays.

[B39] Drenckhahn D, Dermietzel R (1988). Organization of the actin filament cytoskeleton in the intestinal brush border: a quantitative and qualitative immunoelectron microscope study. J Cell Biol.

[B40] Ivanov AI, Bachar M, Babbin BA, Adelstein RS, Nusrat A, Parkos CA (2007). A unique role for nonmuscle myosin heavy chain IIA in regulation of epithelial apical junctions. PLoS ONE.

[B41] Ivanov AI (2008). Actin motors that drive formation and disassembly of epithelial apical junctions. Front Biosci.

[B42] Ma TY, Boivin MA, Ye D, Pedram A, Said HM (2005). Mechanism of TNF-{alpha} modulation of Caco-2 intestinal epithelial tight junction barrier: role of myosin light-chain kinase protein expression. Am J Physiol Gastrointest Liver Physiol.

[B43] Schwarz BT, Wang F, Shen L, Clayburgh DR, Su L, Wang Y, Fu YX, Turner JR (2007). LIGHT signals directly to intestinal epithelia to cause barrier dysfunction via cytoskeletal and endocytic mechanisms. Gastroenterology.

[B44] Barbosa LA, Goto-Silva L, Redondo PA, Oliveira S, Montesano G, De Souza W, Morgado-Diaz JA (2003). TPA-induced signal transduction: a link between PKC and EGFR signaling modulates the assembly of intercellular junctions in Caco-2 cells. Cell Tissue Res.

[B45] Clarke H, Soler AP, Mullin JM (2000). Protein kinase C activation leads to dephosphorylation of occludin and tight junction permeability increase in LLC-PK1 epithelial cell sheets. J Cell Sci.

[B46] Sjo A, Magnusson KE, Peterson KH (2003). Distinct effects of protein kinase C on the barrier function at different developmental stages. Biosci Rep.

[B47] Turner JR, Angle JM, Black ED, Joyal JL, Sacks DB, Madara JL (1999). PKC-dependent regulation of transepithelial resistance: roles of MLC and MLC kinase. Am J Physiol.

[B48] Yoo J, Nichols A, Mammen J, Calvo I, Song JC, Worrell RT, Matlin K, Matthews JB (2003). Bryostatin-1 enhances barrier function in T84 epithelia through PKC-dependent regulation of tight junction proteins. Am J Physiol Cell Physiol.

[B49] Rajasekaran SA, Barwe SP, Gopal J, Ryazantsev S, Schneeberger EE, Rajasekaran AK (2007). Na-K-ATPase regulates tight junction permeability through occludin phosphorylation in pancreatic epithelial cells. Am J Physiol Gastrointest Liver Physiol.

[B50] Rajasekaran SA, Gopal J, Espineda C, Ryazantsev S, Schneeberger EE, Rajasekaran AK (2004). HPAF-II, a cell culture model to study pancreatic epithelial cell structure and function. Pancreas.

[B51] Bogi K, Lorenzo PS, Acs P, Szallasi Z, Wagner GS, Blumberg PM (1999). Comparison of the roles of the C1a and C1b domains of protein kinase C alpha in ligand induced translocation in NIH 3T3 cells. FEBS Lett.

[B52] Bogi K, Lorenzo PS, Szallasi Z, Acs P, Wagner GS, Blumberg PM (1998). Differential selectivity of ligands for the C1a and C1b phorbol ester binding domains of protein kinase Cdelta: possible correlation with tumor-promoting activity. Cancer Res.

[B53] Brose N, Rosenmund C (2002). Move over protein kinase C, you've got company: alternative cellular effectors of diacylglycerol and phorbol esters. J Cell Sci.

[B54] Liu WS, Heckman CA (1998). The sevenfold way of PKC regulation. Cell Signal.

[B55] Webb BL, Hirst SJ, Giembycz MA (2000). Protein kinase C isoenzymes: a review of their structure, regulation and role in regulating airways smooth muscle tone and mitogenesis. Br J Pharmacol.

[B56] Toullec D, Pianetti P, Coste H, Bellevergue P, Grand-Perret T, Ajakane M, Baudet V, Boissin P, Boursier E, Loriolle F (1991). The bisindolylmaleimide GF 109203X is a potent and selective inhibitor of protein kinase C. J Biol Chem.

[B57] Way KJ, Chou E, King GL (2000). Identification of PKC-isoform-specific biological actions using pharmacological approaches. Trends Pharmacol Sci.

[B58] Martiny-Baron G, Kazanietz MG, Mischak H, Blumberg PM, Kochs G, Hug H, Marme D, Schachtele C (1993). Selective inhibition of protein kinase C isozymes by the indolocarbazole Go 6976. J Biol Chem.

[B59] Gschwendt M, Muller HJ, Kielbassa K, Zang R, Kittstein W, Rincke G, Marks F (1994). Rottlerin, a novel protein kinase inhibitor. Biochem Biophys Res Commun.

[B60] Saraiva L, Fresco P, Pinto E, Goncalves J (2003). Isoform-selectivity of PKC inhibitors acting at the regulatory and catalytic domain of mammalian PKC-alpha, -betaI, -delta, -eta and -zeta. J Enzyme Inhib Med Chem.

[B61] Conti MA, Adelstein RS (2008). Nonmuscle myosin II moves in new directions. J Cell Sci.

[B62] Matsumura F (2005). Regulation of myosin II during cytokinesis in higher eukaryotes. Trends Cell Biol.

[B63] Somlyo AP, Somlyo AV (2003). Ca2+ sensitivity of smooth muscle and nonmuscle myosin II: modulated by G proteins, kinases, and myosin phosphatase. Physiol Rev.

[B64] Straight AF, Cheung A, Limouze J, Chen I, Westwood NJ, Sellers JR, Mitchison TJ (2003). Dissecting temporal and spatial control of cytokinesis with a myosin II Inhibitor. Science.

[B65] Kawamoto S, Bengur AR, Sellers JR, Adelstein RS (1989). In situ phosphorylation of human platelet myosin heavy and light chains by protein kinase C. J Biol Chem.

[B66] Moy AB, Blackwell K, Wang N, Haxhinasto K, Kasiske MK, Bodmer J, Reyes G, English A (2004). Phorbol ester-mediated pulmonary artery endothelial barrier dysfunction through regulation of actin cytoskeletal mechanics. Am J Physiol Lung Cell Mol Physiol.

[B67] de Rooij J, Kerstens A, Danuser G, Schwartz MA, Waterman-Storer CM (2005). Integrin-dependent actomyosin contraction regulates epithelial cell scattering. J Cell Biol.

[B68] Samarin SN, Ivanov AI, Flatau G, Parkos CA, Nusrat A (2007). Rho/Rho-associated kinase-II signaling mediates disassembly of epithelial apical junctions. Mol Biol Cell.

[B69] Verin AD, Liu F, Bogatcheva N, Borbiev T, Hershenson MB, Wang P, Garcia JG (2000). Role of ras-dependent ERK activation in phorbol ester-induced endothelial cell barrier dysfunction. Am J Physiol Lung Cell Mol Physiol.

[B70] Leung T, Manser E, Tan L, Lim L (1995). A novel serine/threonine kinase binding the Ras-related RhoA GTPase which translocates the kinase to peripheral membranes. J Biol Chem.

[B71] Matsui T, Amano M, Yamamoto T, Chihara K, Nakafuku M, Ito M, Nakano T, Okawa K, Iwamatsu A, Kaibuchi K (1996). Rho-associated kinase, a novel serine/threonine kinase, as a putative target for small GTP binding protein Rho. EMBO J.

[B72] Aktories K, Mohr C, Koch G (1992). Clostridium botulinum C3 ADP-ribosyltransferase. Curr Top Microbiol Immunol.

[B73] Song JC, Hanson CM, Tsai V, Farokhzad OC, Lotz M, Matthews JB (2001). Regulation of epithelial transport and barrier function by distinct protein kinase C isoforms. Am J Physiol Cell Physiol.

[B74] D'Souza T, Indig FE, Morin PJ (2007). Phosphorylation of claudin-4 by PKCepsilon regulates tight junction barrier function in ovarian cancer cells. Exp Cell Res.

[B75] Bogatcheva NV, Verin AD, Wang P, Birukova AA, Birukov KG, Mirzopoyazova T, Adyshev DM, Chiang ET, Crow MT, Garcia JG (2003). Phorbol esters increase MLC phosphorylation and actin remodeling in bovine lung endothelium without increased contraction. Am J Physiol Lung Cell Mol Physiol.

[B76] Kolosova IA, Ma SF, Adyshev DM, Wang P, Ohba M, Natarajan V, Garcia JG, Verin AD (2004). Role of CPI-17 in the regulation of endothelial cytoskeleton. Am J Physiol Lung Cell Mol Physiol.

[B77] Srinivas SP, Satpathy M, Guo Y, Anandan V (2006). Histamine-induced phosphorylation of the regulatory light chain of myosin II disrupts the barrier integrity of corneal endothelial cells. Invest Ophthalmol Vis Sci.

[B78] Garcia JG, Davis HW, Patterson CE (1995). Regulation of endothelial cell gap formation and barrier dysfunction: role of myosin light chain phosphorylation. J Cell Physiol.

[B79] Chiba H, Kojima T, Osanai M, Sawada N (2006). The significance of interferon-gamma-triggered internalization of tight-junction proteins in inflammatory bowel disease. Sci STKE.

[B80] Turner JR (2006). Molecular basis of epithelial barrier regulation: from basic mechanisms to clinical application. Am J Pathol.

[B81] Riento K, Ridley AJ (2003). Rocks: multifunctional kinases in cell behaviour. Nat Rev Mol Cell Biol.

[B82] Yoneda A, Multhaupt HA, Couchman JR (2005). The Rho kinases I and II regulate different aspects of myosin II activity. J Cell Biol.

[B83] Yoneda A, Ushakov D, Multhaupt HA, Couchman JR (2007). Fibronectin matrix assembly requires distinct contributions from Rho kinases I and -II. Mol Biol Cell.

[B84] Clarke H, Marano CW, Peralta Soler A, Mullin JM (2000). Modification of tight junction function by protein kinase C isoforms. Adv Drug Deliv Rev.

[B85] Barandier C, Ming XF, Rusconi S, Yang Z (2003). PKC is required for activation of ROCK by RhoA in human endothelial cells. Biochem Biophys Res Commun.

[B86] Akopov SE, Zhang L, Pearce WJ (1998). Regulation of Ca2+ sensitization by PKC and rho proteins in ovine cerebral arteries: effects of artery size and age. Am J Physiol.

[B87] Strassheim D, May LG, Varker KA, Puhl HL, Phelps SH, Porter RA, Aronstam RS, Noti JD, Williams CL (1999). M3 muscarinic acetylcholine receptors regulate cytoplasmic myosin by a process involving RhoA and requiring conventional protein kinase C isoforms. J Biol Chem.

[B88] Chang J, Xie M, Shah VR, Schneider MD, Entman ML, Wei L, Schwartz RJ (2006). Activation of Rho-associated coiled-coil protein kinase 1 (ROCK-1) by caspase-3 cleavage plays an essential role in cardiac myocyte apoptosis. Proc Natl Acad Sci USA.

[B89] Sapet C, Simoncini S, Loriod B, Puthier D, Sampol J, Nguyen C, Dignat-George F, Anfosso F (2006). Thrombin-induced endothelial microparticle generation: identification of a novel pathway involving ROCK-II activation by caspase-2. Blood.

[B90] Sebbagh M, Hamelin J, Bertoglio J, Solary E, Breard J (2005). Direct cleavage of ROCK II by granzyme B induces target cell membrane blebbing in a caspase-independent manner. J Exp Med.

[B91] Sebbagh M, Renvoize C, Hamelin J, Riche N, Bertoglio J, Breard J (2001). Caspase-3-mediated cleavage of ROCK I induces MLC phosphorylation and apoptotic membrane blebbing. Nat Cell Biol.

[B92] Ivanov AI, Hopkins AM, Brown GT, Gerner-Smidt K, Babbin BA, Parkos CA, Nusrat A (2008). Myosin II regulates the shape of three-dimensional intestinal epithelial cysts. J Cell Sci.

[B93] Minamide LS, Shaw AE, Sarmiere PD, Wiggan O, Maloney MT, Bernstein BW, Sneider JM, Gonzalez JA, Bamburg JR (2003). Production and use of replication-deficient adenovirus for transgene expression in neurons. Methods Cell Biol.

