# Supplementary material for: Protein kinase C activation disrupts epithelial apical junctions via ROCK-II dependent stimulation of actomyosin contractility
Source: BMC Cell Biol. 2009 May 7;10:36. doi: 10.1186/1471-2121-10-36 (PMC2685374; doi:10.1186/1471-2121-10-36)
Supplement: Additional file 1 — Detection of different PKC isoforms in HPAF-II cells. The data provided represent the RT-PCR analysis of different PKC isoform expression in confluent HPAF-II cell monolayers. (A) Agarose electrophoresis of PCR amplicons shows expression of two classical (PKCs α and γ) and four novel (PKCs δ, ε, η and θ) PKC isoforms. (B) Primer sequences that were used to detect expression of different PKC isoenzymes. [file 1471-2121-10-36-S1.pdf]

A

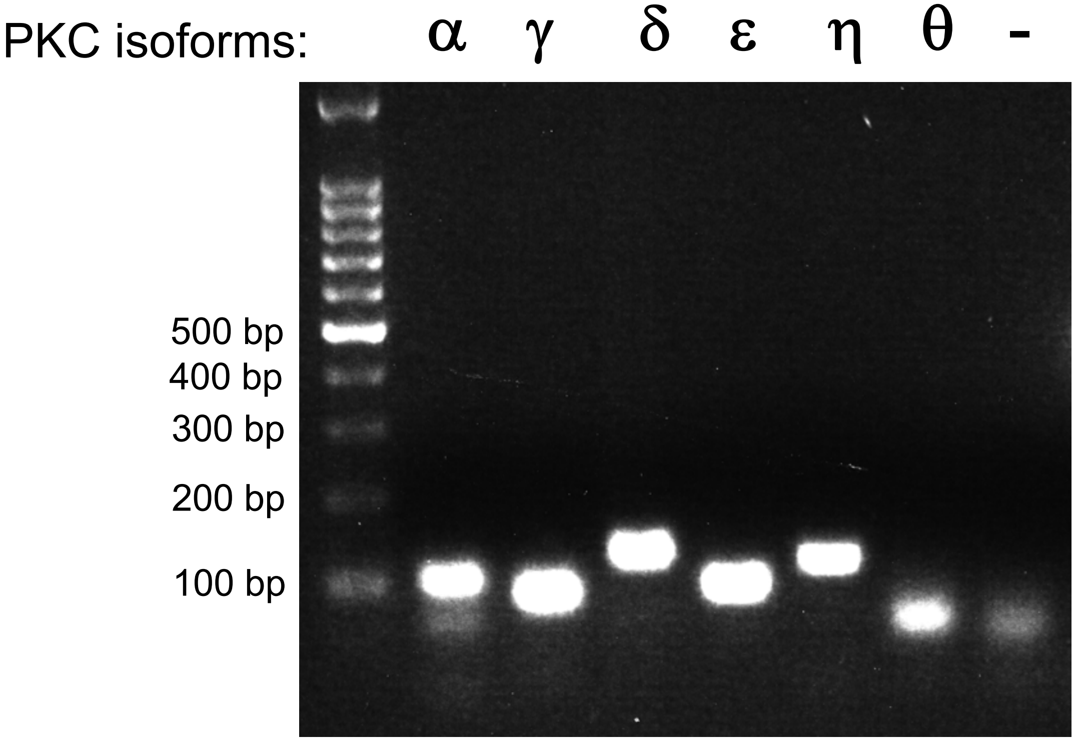

B

Sequences of primes used for amplification of different PKC isoforms

Target gene	Forward primer	Reverse primer	Expected product size, bp
PKCα	TTTGGAAAGGTGATGCTTGCCGAC	TGCACTCCACGTCATCATCCTGAA	103
PKCγ	AAGACTTGAGCGGAGCCCGATATT	ATGGTTAGTGGTGTGGTCTCTGGA	86
PKCδ	TGCCGCTGAGATAATGTGTGGACT	GCACATCCCAAAGTCGGCAATCTT	127
PKCε	CAACCAAGCAAGCTCTAACCGCAA	TTGTCCTGTAGGAAAGGCCCAGTT	94
PKCη	TGAAGGTGCTGAAGAAGGACGTGA	AGCAGCAGAACAACTGAGTGAGGA	120
PKCθ	TAATGACGATGCCGTGTGTCTCGT	AGTGCGGAGACCCATCTTTCAAGT	106
