# Supplementary figures and images for: Protein kinase C activation disrupts epithelial apical junctions via ROCK-II dependent stimulation of actomyosin contractility
Source: BMC Cell Biol. 2009 May 7;10:36. doi: 10.1186/1471-2121-10-36 (PMC2685374; doi:10.1186/1471-2121-10-36)

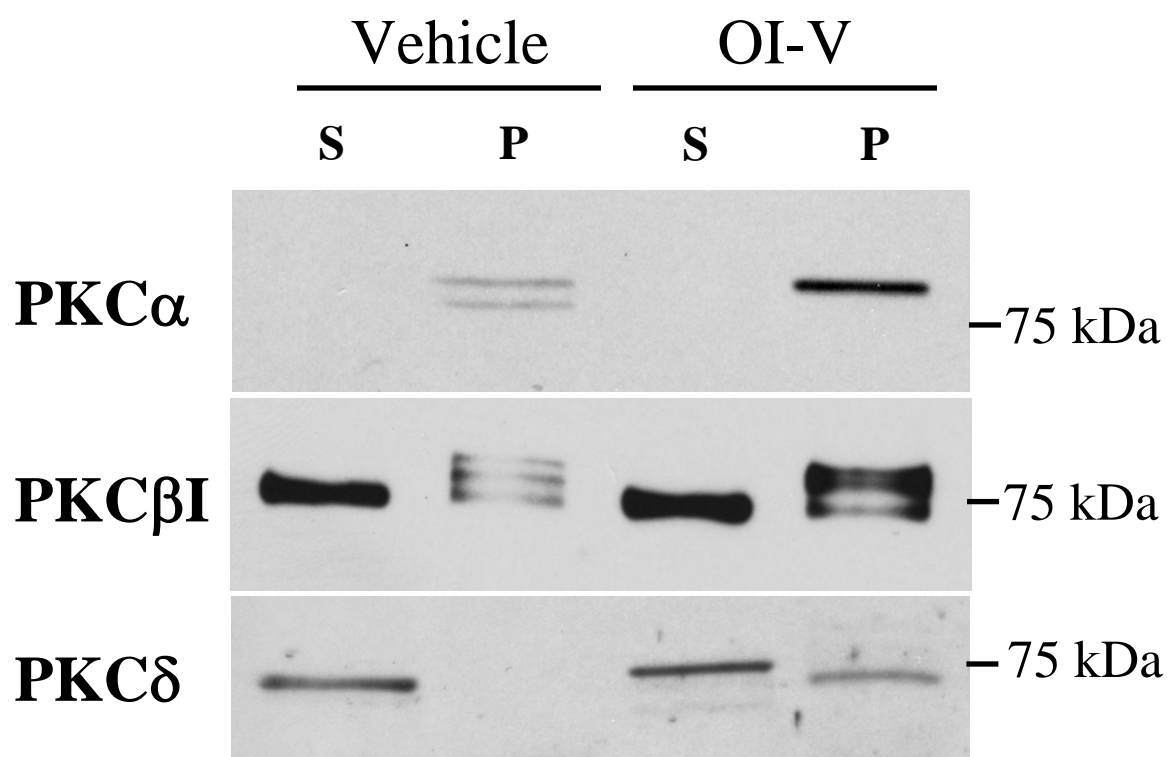

Supplement: Additional file 2 — OI-V induces membrane translocation of different PKC isoforms. The data presented show distribution of different PKC isoenzymes between cytosolic and plasma membrane fractions in control and OI-V-treated epithelial cells. Note that 1 h OI-V treatment rapidly increased the amount of both classical (α and βI) and novel (δ) PKC isoenzymes associated with cell membranes. [file 1471-2121-10-36-S2.pdf]

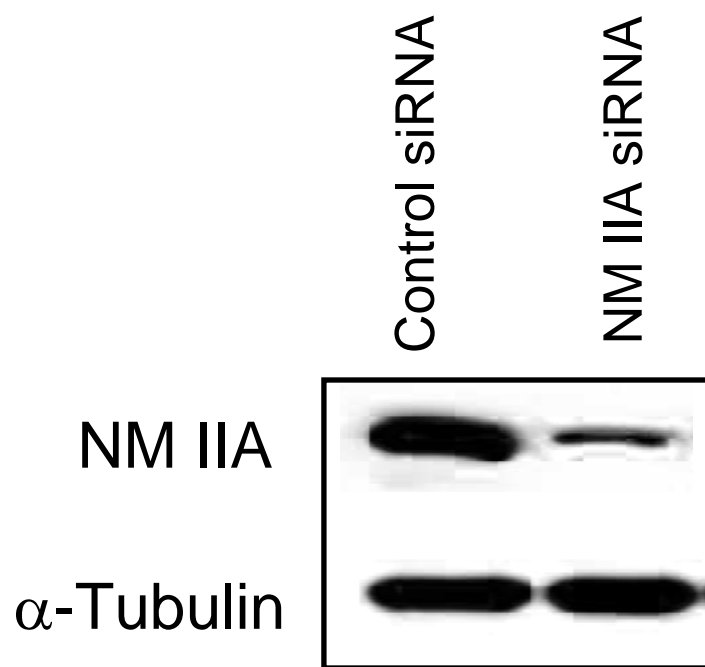

Supplement: Additional file 3 — NM IIA-specific siRNAs effectively downregulate expression of this protein. The data show the effectiveness of siRNA-mediated depletion of NM IIA. HPAF-II cells were transfected with either a control (cyclophilin B), or NM IIA-specific siRNA SmartPools and analyzed for NM IIA expression 84 h post-transfection. [file 1471-2121-10-36-S3.pdf]

**A**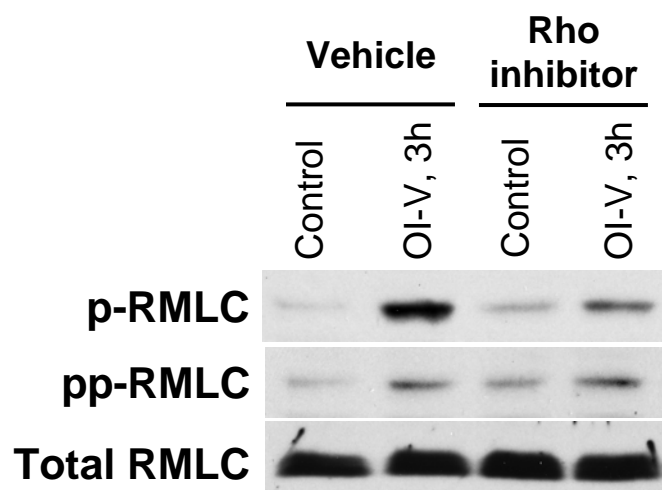**B**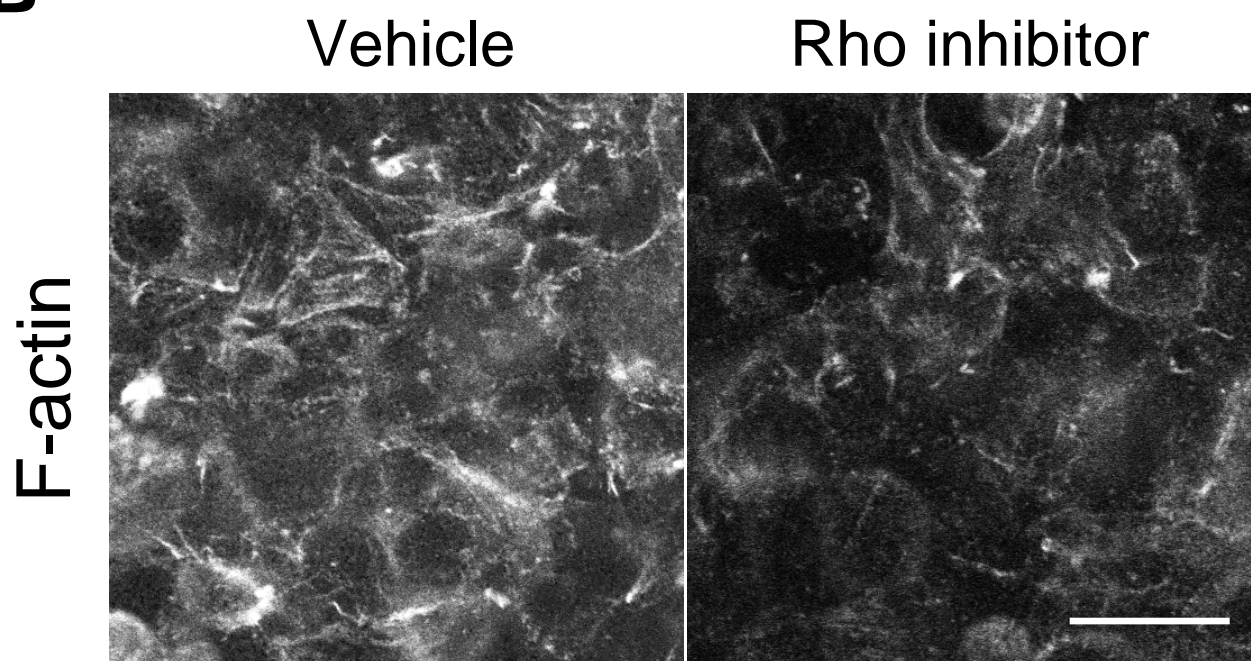

Supplement: Additional file 4 — Effects of Rho inhibition on myosin phosphorylation and basal F-actin filaments in OI-V-treated and control epithelial cells. The presented data demonstrate different effects of Rho inhibition on actomyosin cytoskeleton in HPAF-II cells. (A) HPAF-II cells were treated for 3 h with either OI-V alone (1 μM), or in a combination with cell-permeable Rho inhibitor, C3 toxin (2 μg/ml). Immunoblotting analysis shows that Rho inhibitor fails to prevent OI-V-dependent increase in the amount of mono- and di-phosphorylated RMLC. (B) Control HPAF-II monolayers were treated for 3 h with either vehicle or C3 toxin with subsequent fixation and fluorescence labeling of F-actin. Note that Rho inhibitor causes disappearance of basal actin filaments in HPAF-II cell monolayers which indicates the efficiency of this inhibitor in our experimental conditions. Bar, 20 μm. [file 1471-2121-10-36-S4.pdf]

**A**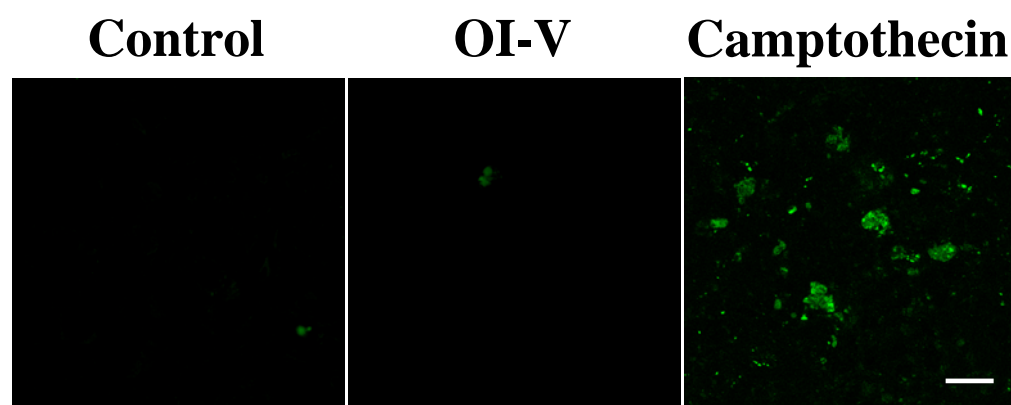**B**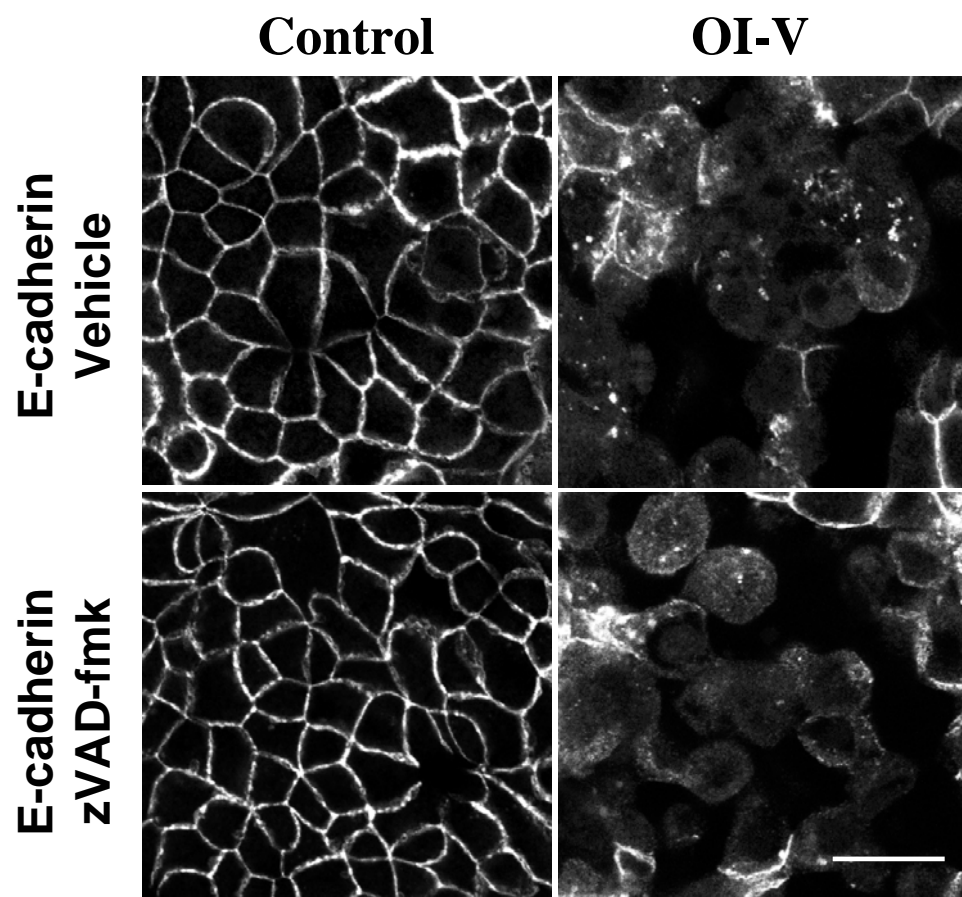

Supplement: Additional file 5 — OI-V-induced junctional disassembly is independent of apoptosis. These experiments probed the role of caspase activation in OI-V-induced junctional disassembly (A) Confluent HPAF-II cell monolayers were incubated for 5 h with either vehicle, or OI-V. Cell monolayers exposed for 5 h to a classical pro-apoptotic agent, camptothecin (2 μg/mL), were used as a positive control. Cell monolayers were fixed and probed with Poly Caspase FLICA detection kit. Note, that camptothecin-treated cells show a significant increase in the number of FLICA-positive caspase-activated cells (green), whereas OI-V does not induce such a caspase activation. (B) HPAF-II cell monolayers were treated for 3 h with either vehicle or 1 μM OI-V with or without pretreatment with a potent pan-caspase inhibitor zVAD-fmk (50 μM). Junctional integrity was evaluated by immunolabeling for E-cadherin. Note that caspase inhibition has no effect on AJ disassembly induced by OI-V. Bar, 20 μm. [file 1471-2121-10-36-S5.pdf]
